# The First Metabolome Analysis in Children with Epilepsy and ALG13-CDG Resulting from c.320A>G Variant

**DOI:** 10.3390/children8030251

**Published:** 2021-03-23

**Authors:** Justyna Paprocka, Aleksandra Jezela-Stanek, Łukasz Boguszewicz, Maria Sokół, Patryk Lipiński, Ewa Jamroz, Ewa Emich-Widera, Anna Tylki-Szymańska

**Affiliations:** 1 Department of Pediatric Neurology, Faculty of Medical Sciences in Katowice, Medical University of Silesia, 40-055 Katowice, Poland; jamroz.ewa5@gmail.com (E.J.); marekwidera@wp.pl (E.E.-W.); 2 Department of Genetics and Clinical Immunology, National Institute of Tuberculosis and Lung Diseases, 01-138 Warsaw, Poland; jezela@gmail.com; 3 Department of Medical Physics, Maria Sklodowska-Curie National Research Institute of Oncology, 44-102 Gliwice, Poland; Lukasz.Boguszewicz@io.gliwice.pl (Ł.B.); Maria.Sokol@io.gliwice.pl (M.S.); 4 Department of Pediatrics, Nutrition and Metabolic Disorders, Children’s Memorial Health Institute, 04-730 Warsaw, Poland; P.Lipinski@ipczd.pl (P.L.); A.Tylki@ipczd.pl (A.T.-S.)

**Keywords:** ALG13-CDG, c.320A>G variant (p.Asn107Ser), epilepsy, metabolome

## Abstract

Background: ALG13-CDG belongs to the congenital disorders of glycosylation (CDG), which is an expanding group of multisystemic metabolic disorders caused by the N-linked, O-linked oligosaccharides, shared substrates, glycophosphatidylinositol (GPI) anchors, and dolichols pathways with high genetic heterogeneity. Thus, as far as clinical presentation, laboratory findings, and treatment are concerned, many questions are to be answered. Three individuals presented here may serve as a good example of clinical heterogeneity. This manuscript describes the first metabolomic analysis using NMR in three patients with epileptic encephalopathy due to the recurrent c.320A>G variant in *ALG13,* characterized to date only in about 60 individuals (mostly female). This is an important preliminary step in the understanding of the pathogenesis of the disease associated with this variant in the rare genetic condition. The disease is assumed to be a disorder of N-glycosylation given that this is the only known function of the ALG13 protein. Despite this, protein electrophoresis, which is abnormal in most conditions due to abnormalities in N-glycosylation, has been normal or only mildly abnormal in the ALG13 patients. Methods: Nuclear magnetic resonance (NMR) spectroscopy in conjunction with multivariate and univariate modelling were used to analyze the metabolic profile of the blood serum samples acquired from the studied patients. Results: Three metabolites were identified as potential biomarkers: betaine, N-acetyl-glycoprotein, and carnitine. Conclusions: Since presented data are the first to be collected so far, they need be verified in further studies. Our intention was to turn attention toward possible CDG-ALG13 laboratory markers that would have clinical significance.

## 1. Introduction

Glycosylation is the most common post-translational modification of proteins and lipids, and its biological role is crucial in the processes of development, growth, and functioning of the body. More than 140 different congenital disorders of glycosylation (CDG) types associated with abnormal protein glycosylation are currently described [1,2,3,4,5,6]. According to the current nomenclature, CDG can be classified into defects in protein N-glycosylation, O-glycosylation, glycosphingolipid and glycosylphosphatidylinositol anchor glycosylation defects, and multiple glycosylation pathways defects. N-glycosylation defects are responsible for more than 70 types [1,2,3,4,5,6]. The basic test in the diagnosis of abnormal protein N-glycosylation is the determination of transferrin isoforms by electrofocusing. An abnormal profile of cathodic shift isoforms is a biochemical marker of congenital glycosylation disorders. However, the protein electrophoresis, which is abnormal in most conditions due to abnormalities in N-glycosylation, has been normal or only mildly abnormal in the ALG13-CDG patients [7]. Clinical presentation and course of CDG are incredibly variable, from the global developmental delay with epilepsy and brain malformations to subclinical coagulopathy [1,2,3,4,5,6]. Given the clinical heterogeneity, the highest diagnostic yield for CDG is broadened genetic testing using next-generation-based methodology, especially whole-exome sequencing (WES) or clinical exome sequencing (CES). They allow for diagnosis and definition of the CDG type, including ALG13 type.

ALG13-CDG presents as an early infantile epileptic encephalopathy. According to the most recent data, the c.320A>G (p.Asn107Ser) variant is the most frequent female predominant mutation [8,9,10,11,12,13,14]. The clinical courses are, however, very variable among affected individuals, which justifies and explains the need to look for new markers or risk factors related to the phenotype.

This paper describes the first metabolomic analysis using proton high resolution nuclear magnetic resonance (1H HR NMR) spectroscopy in three patients with epileptic encephalopathy due to the recurrent c.320A>G variant in *ALG13*. The metabolome characteristics of these three female patients are presented, giving assumptions for further studies that could verify the role of three metabolites as biomarkers suitable for clinical or treatment assessment. As we believe, it is an important preliminary step in the understanding of the pathogenesis of the disease associated with this variant. It is also a very first approach to employ NMR techniques in CDG studies. This is an important preliminary step in the understanding of the pathogenesis of the disease associated with this variant, which has been seen in nearly 60 patients worldwide (mostly female).

## 2. Subjects and Methods

### 2.1. Patients

The study was approved by the ethical committee of the Silesian Medical University, and written informed consent was obtained from each child’s parent(s) or guardian for the participation of their child in the study. All methods were performed in accordance with the relevant guidelines and regulations.

Three female probands with de novo c.320A>G variant in the *ALG13* gene are described (Patients 1–3, referred to as Study Group, SG). Patient 3 was also heterozygous for c.3433C>T (Leu1145Phe;) in the *CACNA1A* gene, which is of unknown significance; its potential impact on the clinical state is thus impossible to define. The age of the patients at the last visit ranged from 2 to 5 years 8 months.

Patient 1

The patient was the second child of nonconsanguineous Polish parents born from an uneventful pregnancy at 38 weeks of gestation with a birth mass of 2430 g. Since birth, epileptic seizures were observed in the form of myoclonic seizures and facial grimaces. Valproic acid and vigabatrin treatment was used. At the age of 8 months of age, she presented with an axial hypotonia and psychomotor retardation. MRI of the brain was normal with myelination appropriate for the patient’s age. Hypsarrhythmia was observed in EEG pattern.

At the last follow-up at 2 years of age, the child presented with microcephaly and developmental delay, but slow improvement was observed (the child sat independently, did not walk, said few words). A visual impairment was diagnosed in the form of astigmatism and hyperopia. No seizures were observed. The monotherapy with valproic acid was continued.

Patient 2

The patient was the second child of nonconsanguineous Polish parents born from an uneventful pregnancy at 41 weeks of gestation with a birth mass of 4510 g. Since the age of 4 months, epileptic seizures were observed, firstly as classical tonic-clonic seizures (treated with vigabatrin), then as myoclonic seizures (treated with valproic acid). Since the age of 6 months, the aggravation of seizures was observed; thus, ACTH (adrenocorticotropic hormone) treatment was implemented. The child at that time presented with an axial hypotonia, a developmental delay, and microcephaly. Hypsarrhythmia was observed in the EEG pattern. MRI of the brain revealed slightly enlarged lateral ventricles with myelination appropriate for the patient’s age. There was no improvement in epileptic seizures. Topiramate was introduced and then switched to lamotrigine. Drug-resistant epilepsy was finally diagnosed.

At the last follow-up at 4 years of age, the child presented with microcephaly and severe global developmental delay, but slow improvement in motor skills were observed (the child sat independently, did not walk). A visual impairment was diagnosed in the form of astigmatism and hyperopia. Seizures were observed every day or every second day.

Patient 3

The patient was the second child of nonconsanguineous Polish parents born from pregnancy complicated by gestational diabetes at 38 weeks of gestation with a birth mass of 3320 g. A spontaneous miscarriage of a first pregnancy at 8–10 weeks of gestation was observed. Since the age of 4 months, myoclonic seizures were observed. At 6 months of age, the child presented with opisthotonos, developmental delay, strabismus, and relative macrocephaly (OFC 90–97pc). Hypsarrhythmia was observed in the EEG pattern. MRI of the brain was normal with myelination appropriate for the patient’s age. In the next several months, the aggravation of seizures, myoclonic in form, was observed; thus, lamotrigine, and then vigabatrin, and finally ACTH treatment were implemented.

At the last follow-up at 5 years and 8 months of age, the child presented with relative macrocephaly (OFC 90–97pc), a severe global developmental delay, but a slow improvement of the motor skills was observed (the child sat independently, did not walk). The seizures were observed every day or every second day. The current treatment includes valproic acid, topiramate, and levetiracetam.

### 2.2. Metabolomic Analyses

Nuclear magnetic resonance (NMR) spectroscopy in conjunction with multivariate and univariate modelling were used to analyze the metabolic profile of the blood serum samples acquired from the studied patients. The obtained metabolic profiles were then compared with the ones from the pediatric drug-resistant epilepsy patients (EG) as well as from the pediatric reference group (RG). The characteristics of the EG and RG groups and the detailed description of the blood samples collection, as well as the NMR acquisition protocol, are described in [15], and a brief summary is given below.

The EG group consisted of 28 patients suffering from drug-resistant epilepsy (the median age was 12 months, the female-to-male ratio was 13:15). The types of seizures and epilepsy as well as the epilepsy syndromes were defined in accordance with the International League Against Epilepsy Classification and Terminology (ILAE 2017). All EG patients underwent diagnostic protocol including EEG, videoEEG, 24-hour EEG (in selected sapienti), laboratory tests for inborn errors of metabolism, chromosomal analysis, and molecular study. The RG group included 20 patients (the median age was 20 months, the female-to-male ratio was 7:13). All children were free of neurological symptoms, developmental delay, or chronic diseases. The routine laboratory tests, EEG, and MRI of the brain showed no abnormalities.

The ALG13 group consisted of three patients (the patient characteristics are provided in Section 2.1). Per each patient, one serum sample was collected.

The sera samples (500 µl) from the overnight peripheral blood were mixed with 500 µL of phosphate buffer (pH 7.4) with D2O and TSP, and the aliquots of 600 µL were transferred into 5 mm NMR tubes (Wilmad-Labglass, Vineland, NJ, WG-1235–7) and stored at 4 °C until the spectroscopic analysis. 1H NMR spectra were acquired on a Bruker 400 MHz Avance III spectrometer (Bruker Biospin, Rheinstetten, Germany) at the temperature of 310 K and with a constant receiver gain (90.5). NOESY (nuclear Overhauser effect spectroscopy), CPMG (Carr–Purcell–Meiboom–Gill), DIFF (diffusion edited), and two-dimensional JRES (J-resolved) spectra were acquired for each blood serum sample. After automatic post-processing, the spectra were aligned to the alanine signal at 1.5 ppm and bucketed (with a bucket size of 0.02 ppm) over the region between 0.5 and 9.0 ppm with the exclusion of the water residual signal. No normalization was applied. The NMR-visible metabolites were quantified based on the 1D positive projections of the JRES spectra, and the peaks were integrated by area using AMIX (Bruker Biospin, Rheinstetten, Germany). The 1D positive projections of the JRES spectra were used for quantification of low molecular weight metabolites, while the lipid signals were quantified based on the diffusion edited spectra. The peaks were integrated by area using AMIX (Bruker Biospin, Rheinstetten, Germany), and the integrals were measured in the spectral regions defined individually for each low molecular weight metabolite, whereas in the case of the lipid signals, a 0.12 ppm range around a particular peak was applied. Sixty-eight NMR signals (accounting for 29 unique low molecular weight metabolites and 6 lipid signals) were quantified. The multivariate analyses were performed on the bucketed NMR spectra as well as on all quantified metabolites.

The multivariate tools used in the present study were: unsupervised principal component analysis (PCA) and supervised orthogonal partial least-squares discriminant analysis (OPLS-DA). The main results for the multivariate analyses are presented graphically in two types of plots:

- Scores plot (PCA and OPLS-DA)—used to observe the patterns or class separation in the data.
- S-plot (OPLS-DA)—giving insight into the variables’ importance for the observed class separation. The variables shifted away from the plot origin along the *y* axis (p-corr) had high reliability, while the variables more shifted along the *x* axis showed a greater magnitude of the changes. The ideal candidate for a biomarker has both high reliability and magnitude.

PCA visualizes the directions of the largest variation in the data. However, these directions may not always reflect the separation between individual classes. In such a situation, supervised methods are useful, in particular OPLS-DA. OPLS-DA models the relation between the data matrix (usually denoted as **X**) and the information about belonging to a given class (denoted in the form of a zero-one **Y** matrix). The orthogonality of the OPLS-DA method means that the separation between classes on the scores plot is always illustrated along the *x* axis, while the intra-group variability is presented along the *y* axis. This markedly facilitates the interpretation of the results. The amount of variation in the data that is correlated to class separation is given by a predictive component, while an orthogonal component gives the amount of uncorrelated variation between **X** and **Y** matrices. An extensive discussion of these multivariate methods and the plots presenting the results is available in [16,17].

The variables for the PCA and OPLS-DA models were Pareto-scaled; the models were validated using internal 7-fold cross-validation. The OPLS-DA model statistical significance was evaluated using ANOVA of the cross-validated residuals (cv-ANOVA) test.

In addition to multivariate methods, a univariate Kruskal–Wallis ANOVA was used (due to non-normal data distribution) to further investigate the statistical differences between the studied groups.

## 3. Results

### Metabolomic Data

**Figure 1:**
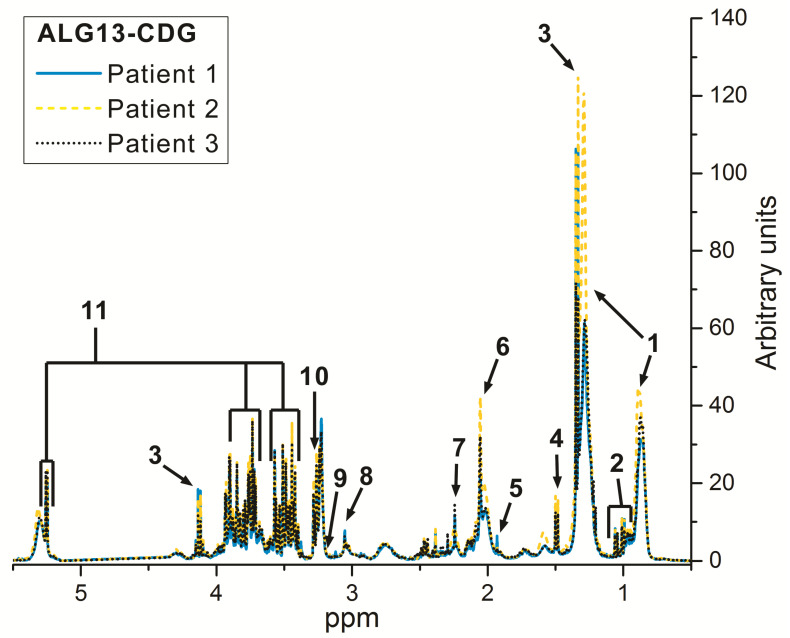
1H-NMR Carr–Purcell–Meiboom–Gill (CPMG) spectra of the serum samples obtained from three *ALG13 c.320A>G* carrier patients: Patient 1—blue line, Patient 2—yellow dashed line, Patient 3—black dotted line. The main detected metabolites are: 1, lipids; 2, BCAA (branched-chain amino-acids); 3, lactate; 4, alanine; 5, acetate; 6, NAG (N-acetyl-glycoprotein); 7, acetone; 8, creatinine; 9, carnitine; 10, betaine; 11, glucose.

Figure 1 shows the 1H-NMR CPMG spectra of the serum samples obtained from three ALG13-CDG patients. The NMR spectra of the EG and RG groups are available in [15].

**Figure 2:**
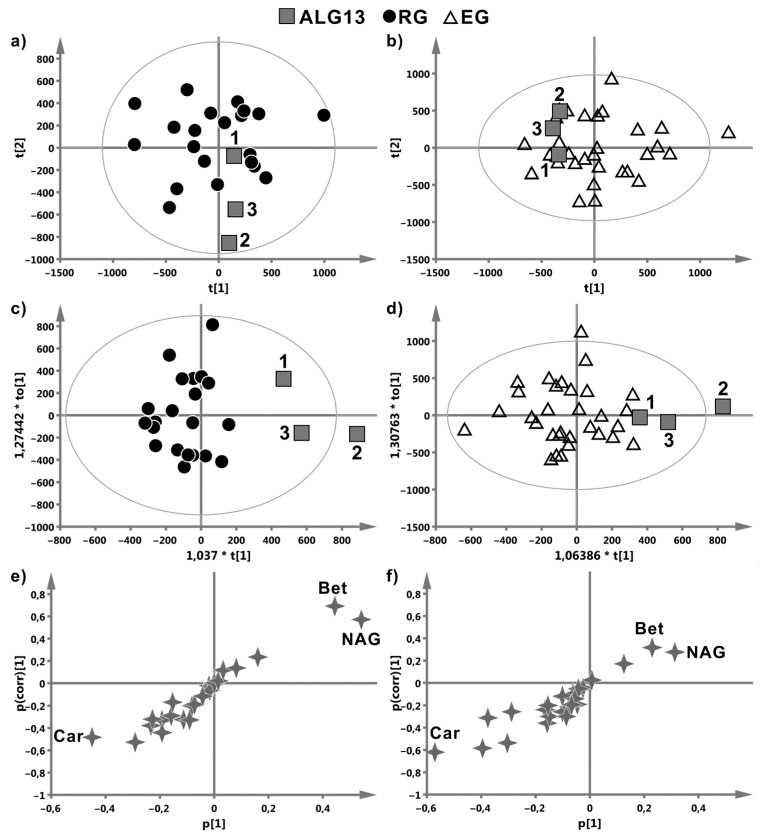
Scores plots from principal component analysis (PCA) (**a**,**b**) and orthogonal partial least-squares discriminant analysis (OPLS-DA) (**c**,**d**) distinguishing the ALG13 cases from reference group (RG) (**a**,**c**) and drug-resistant epilepsy patients group (EG) (**b**,**d**). The *x* and *y* directions in the PCA scores plots (**a**,**b**) reflect the first two directions of the highest variability in the data. In case of subtle metabolic changes these directions may not necessarily correspond well with the class separation. The *x* and *y* directions in the OPLS-DA scores plots (**c**,**d**) correspond to the inter- and intra-class variability, respectively. The metabolites responsible for the clustering observed in the OPLS-DA scores plots are identified based on the OPLS-DA S-plots (**e**,**f**). The further the point is from the center of the plot (along both the *x* and *y* axis), the more impact this variable exerts on the observed class separation in the scores plot (OPLS-DA only).

The multivariate PCA, OPLS-DA models and the S-plots for the ALG13, EG, and RG groups are shown in Figure 2.

The diagnostics of the discussed PCA and OPLS-DA models are provided in Table 1.

The multivariate modelling failed to distinguish the ALG13-CDG cases from the EG group—the resulting OPLS-DA model (Figure 2d,f) showed no statistical significance (cv-ANOVA *p*-value > 0.999, Table 1), while the distinction between ALG13 and RG was close to a statistical significance (cv-ANOVA *p*-value = 0.08, Table 1). Nevertheless, the OPLS-DA models delivered some suggestions about the potential metabolites of interest. Figure 2c,e indicate the increased levels of betaine and N-acetyl-glycoprotein (NAG) as well as the decreased level of carnitine in ALG13 when compared to the RG group.

**Figure 3:**
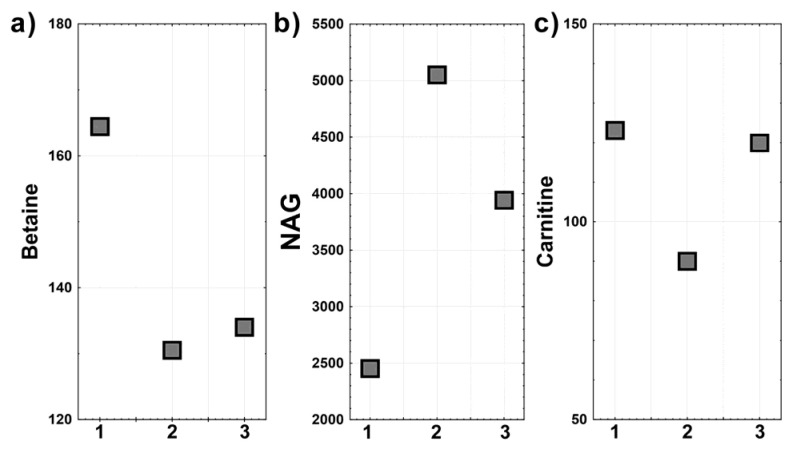
Differences in the relative intensities of betaine (**a**), N-acetyl-glycoprotein (**b**), and carnitine (**c**) among the ALG13 cases: The numbers on the *x* axis correspond to individual ALG13 patients: 1—Patient 1, 2—Patient 2, and 3—Patient 3.

Based on the results from OPLS-DA, we used the scatter plots and the univariate statistics in order to investigate these three metabolites (betaine, NAG, and carnitine) further. Figure 3 presents a scatter plot of the relative intensities of betaine (a), NAG (b), and carnitine (c) for the ALG13-CDG c.320A>G cases. An inverse correlation for the NAG and betaine signals is visible (Patient 1 shows higher betaine and lower NAG when compared to Patients 2 and 3).

**Figure 4:**
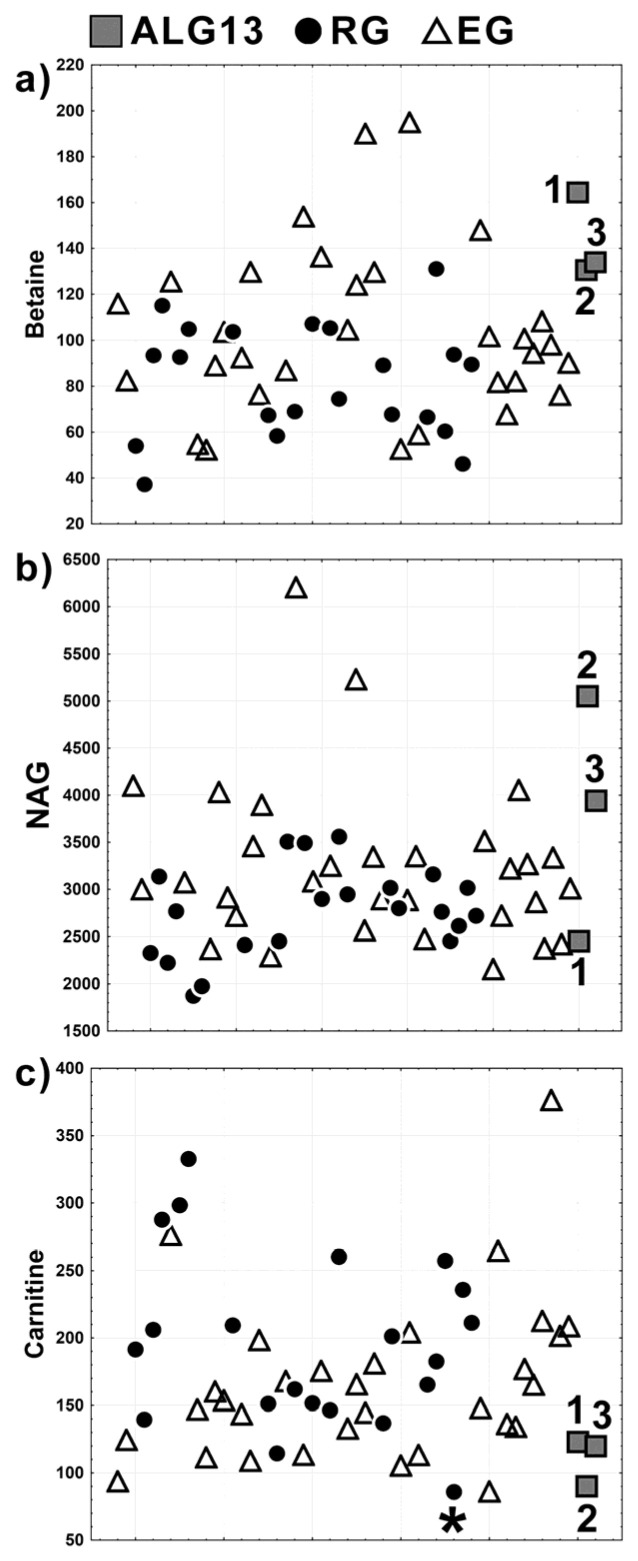
Differences in the relative intensities of betaine (**a**), N-acetyl-glycoprotein (**b**), and carnitine (**c**) among the RG (•), EG (Δ), and ALG13 (▯) groups. The ALG13 cases: 1—Patient 1, 2—Patient 2, and 3—Patient 3. 🞲 denotes the outlying sample from RG due to the lowest level of carnitine in this group. The points in the plot as well as the *x* axis scale correspond to individual patients.

A comparison of the relative intensities of betaine, NAG, and carnitine signals between the ALG13, EG, and RG groups is shown in Figure 4. The statistical differences between the studied groups were assessed using Kruskal–Wallis ANOVA test (Table 2).

It is clearly seen (Figure 4a, Table 2) that betaine was significantly higher in ALG13-CDG when compared to RG (*p*-value = 0.014) and fell in the upper range of variation of the EG group (however, without statistical significance). NAG, in turn, revealed a considerable internal difference in the *ALG13* c.320A>G carrier cases (Figure 4b). For Patient 1 the relative intensity of NAG was extremely low, while in Patients 2 and 3 the NAG signal was higher than in RG and (similarly to betaine) fell into the upper range of variation of the EG group. However, due to the considerable internal variation, the differences were not statistically significant (Table 2). The relative intensity of carnitine showed a very low variability among the ALG13 patients, and it was in the lower range of variation of the EG and RG groups, but without a statistical significance (Figure 4c, Table 2). Yet, after removing one case from the RG group (the case denoted with 🞲 in Figure 4) the difference between the ALG13-CDG c.320A>G and the RG group was still not statistically important; however, the *p*-value for the comparison (*p*-value = 0.074) came closer to significance (Table 2).

Among the metabolites indicated by the OPLS-DA models as the promising biomarkers, only the concentration of carnitine was found to be age-dependent. Thus, the carnitine results presented in Figure 4c and Table 2 were already shown after the correction for age dependency. The age-dependency was determined from the carnitine relative intensities measured for RG, and the correction was made using the residual method for RG, EG, and *ALG13* c.320A>G carriers. The detailed procedure for the age correction is described in detail in [15].

## 4. Discussion

To date, ALG13-CDG has been reported in more than 60 patients, most of which results from c.320A>G variant (*p*.N107S) (about 85%, including 3 males) [6]. This particular mutation causes an unpredictable outcome, as may also be concluded based new female patients described herein (Table 3) and reviewed in detail recently by Ng et al. and Datta et al. [12,14].

Regarding epilepsy, most known ALG13-CDG patients have been treated with a combination of many antiepileptic drugs (AEDs), which unfortunately did not lead to adequate seizures control [13]. Only a few probands have been described as seizures-free, including cases CDG-0134 and CDG-0139 (on a ketogenic diet) or controlled on AED/keto (case CDG-0092) [13]. Moreover, in two other females, the seizures were finally controlled as a result of implemented pharmacotherapy (presented in Table 3) [11,18]. Thus, looking for novel disease biomarkers allowing for clinical and/or treatment monitoring seems to be reasonable.

The presented NMR-based metabolomics results obtained for patients with ALG13-CDG caused by a c.320A>G variant provide an interesting preliminary insight into the laboratory characteristics of one of the glycosylation disorders. The mammalian glycome consists of up to several thousand glycan structures and may be larger even than the proteome. Aberrations in the glycosylation anabolic and catabolic mechanisms may disturb cell adhesion, cell signaling, and endocytosis, with congenital disorders of glycosylation (CDGs) affecting the central and peripheral nervous system, the endocrine system, and coagulation [15,16,19,20]. The metabolic effects of such aberrations can be monitored using NMR-based metabolomics. Quantifying and delineating the human glycome in health and disease has received increasing interest as a novel tool for identifying the markers of disease and the potential mechanistic mediators of disease pathogenesis [17,21].

In our study, the multivariate metabolomic profiling and the descriptive statistics of the NMR spectra of the blood sera revealed that the metabolic alterations presumably characteristic for the *ALG13* c.320A>G carrier cases are restricted to three metabolites: betaine, NAG, and carnitine; however, only betaine was found to be an ALG13 specific marker statistically confirmed by the univariate tests (*p*-values = 0.014 for the comparison with RG). The other two compounds indicated by the OPLS-DA models, carnitine and N-acetyl-glycoprotein, can be only treated as the compounds of a possible importance; however, their role should be confirmed in larger group studies. While the ALG13 and RG groups were clearly separated in the OPLS-DA scores plot (Figure 2c), the model itself was only close to statistical significance (*p*-value = 0.08), presumably due to a small size of the ALG13 group. The separation between ALG13 and EG was markedly weaker (Figure 2d), as reflected by the lack of a statistical significance for the OPLS-DA model (*p*-value > 0.999). In this case, apart from the small ALG13 group size, the shared similarities of the metabolic profiles of both groups also seem to be of importance. This supposition is supported by the results obtained for a model that differentiates between the EG and RG groups for prediction of class membership of ALG13 patients (data not shown). The ALG13 samples included in this classification were identified by this model as EG (Patients 2 and 3) and as ambiguous (Patient 1). For such classification of Patient 1, the mildest spectrum of ALG13-CDG as compared with the other two cases may be responsible.

Betaine, a small amino-acid, with its primary role to serve as a methyl group donor in liver metabolism, is also used as an osmoprotectant in most tissues in the body [18,22]. The blood serum concentration of betaine is mostly regulated by a dietary intake of betaine or the choline rich compounds, but it is also synthesized from choline in the liver and kidney (it is linked to folate-dependent one-carbon metabolism) [19,23]. The relationship between betaine and various diseases has been the subject of intensive research in the last decade, and most of the findings link the lower blood betaine levels with pathology [20,24]. Inversely, in our study, the ALG13 patients showed the highest betaine levels when compared with the RG and EG groups. Plasma betaine level is under homeostatic control, the possible reasons of the elevated betaine in blood are an increased supplementation [20,24], a decreased efficiency of betaine retention by cells (impaired cellular osmoregulation) [21,25], an increased choline oxidation, or impaired metabolism related to homocysteine methyltransferase (BHMT)—betaine is mainly eliminated by metabolism, it is catabolized by BHMT [22,26]. Furthermore, recent publications reveal anti-inflammatory properties of betaine but without information about whether the endogenous betaine increases or decreases during inflammation [18,22].

Glycosylation produces different types of glycans (or glycoconjugates) that are typically attached to cellular proteins and lipids. Approximately half of all proteins typically expressed in a cell undergo glycosylation. There are mainly two categories of glycosylation: N-glycosylation and O-glycosylation. N-glycans are linked to the amide group of asparagine, while O-glycans are linked to the hydroxyl group of serine or threonine [4,23]. One of the three metabolites indicated by OPLS-DA as important for distinction of the ALG13-CDG patients harboring c.320A>G variant was NAG (it is represented mainly by N-acetylglucosamine and N-acetylneuramic acid—the acute phase proteins with anti-inflammatory properties and are expressed more during inflammation and immune responses)—a serum NMR marker for inflammation [24,27]—and an inverse relation between NAG and betaine is visible in Figure 3 and Figure 4. However, the small size of the ALG13 group makes it impossible to infer the relationship between the analyte levels and the clinical severity of the disease in the ALG13-CDG patients. Furthermore, no correlation between NAG and betaine in the RG and EG groups could be found. Because there were only three cases in the ALG13 group, it is impossible to determine whether the NAG-betaine relation is ALG13-specific or only a coincidence. Thus, though NAG seems to be a biomarker of the c.320A>G variant in ALG13 mutant patients, its specificity is still an open question due to a small number of the studied patients. On the other hand, the standard laboratory glycosylation studies performed in patients with the c.320A>G variant in *ALG13* are claimed to be near-normal [8]; thus, the analysis of serum N-glycans using NMR is a more sensitive test and as such provides an added value in CDG research and diagnostics. It is possible that NAG could provide broader profiling of systemic inflammation in the c.320A>G variant in ALG13 owing to the summative characteristics of this signal. Additional prospective studies will be valuable in further assessing the relationship between NAG and clinical outcomes.

The third metabolite identified by OPLS-DA and with close to statistical significance difference between ALG13 and RG (Table 2)—carnitine—showed a relatively low intra-group variability in our ALG13-CDG patients and, contrary to betaine and NAG, is decreased when compared to RG and EG. Carnitine, a non-essential amino acid, is synthesized in the liver and functions as a carrier of long-chain fatty acids across the inner mitochondrial membrane [25,28]. Endogenous carnitine is stored in the cardiac and skeletal muscles, and its concentration in blood should be interpreted in conjunction with other metabolic or tissue specific information [26,29]. Secondary carnitine deficiency is mostly observed in association with the liver or kidney disease, defects in fatty acids metabolism, malnutrition, and administration of valproic acid, as well as during the inflammatory state [4,30]. We suppose that the latter two effects might be responsible for the low carnitine in ALG13. On the other hand, fatty acids, glucose, and glutamine are the substrates (in cooperation with, inter alia, Acetyl-CoA) for the UDP-GlcNac; thus, a link between carnitine and UDP-GlcNac cannot be definitely excluded. However, to the best of our knowledge, a direct relation between betaine, NAG, and carnitine with UDP-GlcNac and/or ALG13 gene is not documented. Likewise, the pathway analysis and the network explorer tools implemented in MetaboAnalyst software did not reveal any connection between these compounds.

The main limitation of the study is the very small size of the ALG13 group. However, due to the extreme rarity of the disease, the three analyzed cases account for 5% of all diagnosed cases worldwide. Another limitation is related to OPLS-DA being prone to overfitting. Keeping this in mind, the results present the OPLS-DA model calculated on the quantified metabolites, which drastically reduced the number of the variables (in comparison to the analysis of the whole NMR spectra) and, at least to a small extent, reduced the risk of overfitting. Furthermore, the model was internally cross-validated as well as verified using univariate statistical methods.

## 5. Conclusions

Betaine, N-acetyl-glycoprotein, and carnitine were indicated by OPLS-DA to be possible biomarkers of ALG13; however, only betaine was confirmed by the univariate ANOVA tests to be specific for ALG13-CDG. Hopefully, further studies performed on a larger ALG13 group could verify the role of these possible markers for classifying ALG13-CDG disease severity or/and monitoring disease treatment with anti-epileptic drugs. Defects in the metabolism of amino acids are claimed to lead to pathology of the nervous system and many other organs [31,32]. Metabolomics seems to be helpful in elucidating the role of glycosylation in CDG caused by the c.320A>G *ALG13* variant, in understanding the complex pathophysiology underlying a glycosylation deficiency, and in establishing new therapeutic approaches.

## Figures and Tables

**Table 1:** Diagnostic parameters of the PCA and OPLS-DA models comparing ALG13 with RG and ALG13 with EG.

ALG13 vs. RG				ALG13 vs. EG			
Principal Component Analysis (PCA)							
Principal Component		*R*^2^X (%)	Q^2^	Principal Component		*R*^2^X (%)	Q^2^
PC1		28	0.004	PC1		22.6	0.044
PC2		20.9	0.045	PC2		18.2	0.071
Orthogonal partial least squares—discriminant analysis (OPLS-DA)							
Predictive component	*R*^2^X (%)	*R* ^2^	Q^2^	Predictive component	*R*^2^X (%)	*R* ^2^	Q^2^
P1	12.3	0.796	0.356	P1	10.2	0.407	−0.32
Orthogonal component	*R*^2^X(o) (%)			Orthogonal component	*R*^2^X(o) (%)		
O1	18.5			O1	18.8		
O2	22.7						
cv-ANOVA for OPLS-DA model		*p*-value = 0.08		cv-ANOVA for OPLS-DA model		*p*-value = >0.999	

*R*^2^X—amount of variation explained by the model components (PCA) or amount of variation in the data that is correlated to class separation (OPLS-DA). *R*^2^—fraction of class membership variation modeled using the data matrix. *R*^2^X(o)—amount of variation uncorrelated to class separation. Q^2^—predictive ability.

**Table 2:** The *p*-values from Kruskal–Wallis ANOVA showing the statistical differences in the relative intensities of betaine, NAG, and carnitine between the RG and EG groups and three ALG13 cases.

*p*-Values from Kruskal–Wallis ANOVA			
Betaine			
	EG	RG	ALG13
EG		0.186	0.132
RG			0.014
ALG13	0.186	0.014	
NAG			
	EG	RG	ALG13
EG		0.175	>0.999
RG	0.175		0.363
ALG13	>0.999	0.363	
Carnitine			
	EG	RG	ALG13
EG		0.251	0.655
RG	0.251		0.13
ALG13	0.655	0.13	
Carnitine after removal of 1 RG case			
EG		0.103	0.62
RG	0.103		0.074
ALG13	0.62	0.074	

**Table 3:** Neurodevelopmental histories of ALG13-CDG patients with c.320A>G variant who did respond to the antiepileptic treatment [11,18] and those diagnosed by us as ALG13 cases.

	Kobayashi et al. 2016 [18]	Madaan et al. 2019 [11]	Patient 1	Patient 2	Patient 3
Age/gender	Female	30 mo	2 yrs/female	4 yrs/female	5 yrs/female
Family history	not reported	not reported	unremarkable	unremarkable	recurrent ischemic strokes, also in the mother 2 times before pregnancy
Gestation (G) and delivery period (D)	not reported	not reported	GII, DII (Cesarean section, of maternal indication) birth weight—2430 g OFC—32 cm birth length—50 cm 10 points in Apgar scale	GII, DII, 42nd week of gestation (green amniotic fluid), 10 points in Apgar scale, normal birth parameters	GII (after months of efforts; DI—miscarriage), complicated by gestational diabetes mellitus, Cesarean section at term, normal birth parameters
Seizures’ morphology	6 mo— epileptic spasms	5 mo— epileptic spasms	4 weeks—epileptic spasms	4 mo—tonic seizures, epileptic spasms	4 mo—epileptic spasms, myoclonic seizures, tonic seizures, partial seizures
EEG	6 mo—hypsarrthythmia	5 mo— hypsarrthythmia	4 weeks- hypsarrthythmia 2 yrs—few generalized paroxysmal changes	4mo— hypsarrthythmia	4 mo—hypsarrthythmia, 5 yrs—partial (L>R) and generalized paroxysmal discharges
Drugs	ACTH	ACTH with epileptic spasms regression for 18mo	Vigabatrin, valproic acid, ACTH at present: valproic acid, reduction vigabatrin dosage	Vigabatrin, valproic acid, ACTH, topiramate, lamotrygine, at present: vigabatrin, lamotrygine	Valproic acid, levetiracetam, ACTH, topiramate, methylprednisolone at present: valproic acid, lamotrygine, topiramate, primidone
Psychomotor development	3yrs—head control	generalized hypotonia, microcephaly, developmental delay	generalized hypotonia, microcephaly, developmental delay; can sit unsupported and crawl; good emotional contact	generalized hypotonia, microcephaly, developmental delay: can sit unsupported, try to walk by the hand; syllabize	generalized severe hypotonia (at 5 yrs is unable to control her head), OFC >97 c, multiple dystonic movements; from birth poor eye contact and lack of interest in the surrounding, 4 mo—noticeable decrease in spontaneous activity
Other complaints/diseases	chorea, dyskinesia	autistic features	astigmatism, strabismus and hyperopia; problems with chewing	tendency to autostimulation and autoaggression; synechia; astigmatism and hypermetropia	strabismus, facial dysmorphism—open-mouth appearance, high forehead, hypertelorism, short and up-turned nose, smooth philtrum and thin vermilion
Brain MRI	cerebral atrophy	normal	slightly delayed myelination of white matter	slight widening of the ventricular system (L>R)	normal

ACTH—adrenocorticotropic hormone.

## Data Availability

The datasets generated during and/or analyzed during the current study are available from the corresponding author on reasonable request.
